# Serum SALL4 Is a Novel Prognosis Biomarker with Tumor Recurrence and Poor Survival of Patients in Hepatocellular Carcinoma

**DOI:** 10.1155/2014/262385

**Published:** 2014-04-17

**Authors:** Su-xia Han, Jun-lan Wang, Xi-jing Guo, Chen-chen He, Xia Ying, Jin-lu Ma, Yuan-yuan Zhang, Qian Zhao, Qing Zhu

**Affiliations:** Department of Medical Oncology, The First Affiliated Hospital of Xi'an Jiaotong University Medical College, Xi'an, Shaanxi 710061, China

## Abstract

*Aim*. Sal-like protein 4 (SALL4), is reexpressed in tissues of a subgroup of HCC associated with poor prognosis. Reports of SALL4 serological levels linked to HCC patients are meager and unclear in the prognosis of this malignancy. *Methods*. Immunohistochemistry and optical microscopy protocols were used to examine the presence of SALL4 in liver tissues from the following patients: 38 HCC, 11 chronic hepatitis B virus (HBV), 13 liver cirrhosis, and 12 healthy controls. Additionally, enzyme-linked immunosorbent assay (ELISA) was used to measure the SALL4 levels in serum samples isolated from patients as follows: 127 with HCC, 27 with HBV, 24 with liver cirrhosis, and 23 normal controls. *Results*. Analysis of liver tissues sections from HCC patients (18 out 38; 47.4%) showed positive staining for SALL4 and its expression did no correlate with any of the clinicopathologic characteristics. HCC patients displayed higher levels (50.4%) of SALL4 protein in serum, compared with the three control groups. Moreover, SALL4 concentration reached the maximum level after one week after treatment and dropped quickly after one month. These HCC patients showing high SALL4 serum levels had poor prognosis, evidenced by both tumor recurrence and overall survival rate. *Conclusions*. High SALL4 serum levels are a novel biomarker in the prognosis of HCC patients.

## 1. Introduction

The latest World Health Organization investigation shows that hepatocellular carcinoma (HCC) is one of the most common malignant diseases and the third leading cause of cancer-related death globally [1, 2]. In 2008, about half of the world's new cases of liver cancer and related-death were from China, of which 70%–80% was HCC. The unique feature of HCC is that antecedent HBV-related chronic hepatitis and liver cirrhosis are common precursor conditions. Due to the lack of sensitive methods for early diagnosis, many patients were diagnosed in advanced disease, missing the best treatment period. Although surgery, chemotherapy, and liver transplantation are effective methods for treatment, the advanced stage of HCC limits their choices for curative treatment [3].

Sal-like protein 4 (SALL4), a zinc finger transcription factor, plays an important role in maintenance of pluripotency and self-renewal of embryonic stem cells by regulating stem cell markers first with NANOG and second with OCT4 and SOX2M [4–6]. SALL4 mutation causes Okihiro syndrome and many researchers have demonstrated that SALL4 is essential in hematopoietic stem cells and regulates their expansion. In primary acute myeloid leukemia (AML) and myelodysplastic syndrome (MDS), the expression of SALL4 is higher than health control. At the meaning time, some other kinds of tumor such as colorectal cancer, breast cancer, and Wilms tumors also express SALL4 [4, 7, 8]. More recently, SALL4 has been suggested as a marker of germ cell tumor, including primary and metastatic of yolk sac tumor [9–13]. In the liver, SALL4 elevated was expressed in fetal liver and was silenced in mature hepatocytes. Some researchers have found that SALL4 was reexpressed and associated with the poorest prognosis [14] in a subgroup of adult hepatocellular carcinomas. However, they had some studies about SALL4 only in HCC tissues without health controls and nonmalignant chronic liver disease. Meanwhile, few studies had been identified that enable the SALL4 serological levels with the prognosis of HCC patients. Thus, in the study, we estimated the expression of SALL4 in HCC tissues and SALL4 serum levels, to explore the possibility to use them as a novel prognostic biomarker in HCC patients.

## 2. Materials and Methods

### 2.1. Liver Tissue and Serum Samples

Total 74 liver tissue sections including 38 patients with HCC, 11 patients with chronic hepatitis B virus (HBV), 13 patients with liver cirrhosis, and 12 healthy controls were obtained from the First Affiliated Hospital of Xi'an Jiaotong University Medical College (Shaanxi Province, China) from 2011 to 2013. All tissue sections were reviewed for confirmation of the original histopathological and clinical diagnosis. All these samples were formalin fixed and paraffin-embedded. The clinicopathologic characteristics of these samples such as tumor size, location, and grade of differentiation were recorded. The clinicopathologic features of patients with HCC are summarized as shown in Table 1. Of 38 HCC patients, 86.8% (33/38) were male and 13.2% (5/38) were female. The mean age was 52.65 ± 12.47 years old. Mean tumor size was 6.18 ± 2.89 cm. 15.8% (6/38) nodal metastasis and 31.6% (12/38) of HCC patients had vascular invasion.

Samples of 201 human sera from 127 patients with HCC, 27 patients with chronic hepatitis, 24 patients with liver cirrhosis, and 23 normal controls were obtained from the First Affiliated Hospital of Xi'an Jiaotong University Medical College (Shaanxi Province, China) from 2011 to 2013. These 127 HCC patients had also been followed up for two years. Overall survival of HCC patients was calculated as the time from diagnosis to the date of death. Patients who were alive were treated as censored for overall survival analysis.

The study was approved by the Institutional Review Board of Xi'an Jiaotong University and collaborating institutions. All patients signed informed consent forms. All patients had not received preoperative chemotherapy, radiotherapy, and embolization and were classified according to the criteria of American Joint Committee on Cancer stage Staging Manual, Seventh Edition (2010) for HCC.

### 2.2. Immunohistochemical Staining Assay

For each analyzed specimen, one or two formalin-fixed paraffin embedded tissue blocks were retrieved to generate 4-5 *μ*m sections for immunohistochemical labeling with antibodies against SALL4 (1 : 100, Clone 6E3; Abnova Corporation, Taipei, Taiwan). Appropriate negative and positive controls were added in each run of immunohistochemistry assay. The primary antibody was detected using biotinylated secondary antibodies (Zhongshan Golden Bridge Biotechnology Ltd. Co., China) according to the manufacturer's recommendations. The final labeling of the tissue sections was performed using the HRP-streptavidin conjugates. The sections were incubated with diaminobenzidine and counterstained with hematoxylin and visualized via optical microscopy.

**Figure 1:**
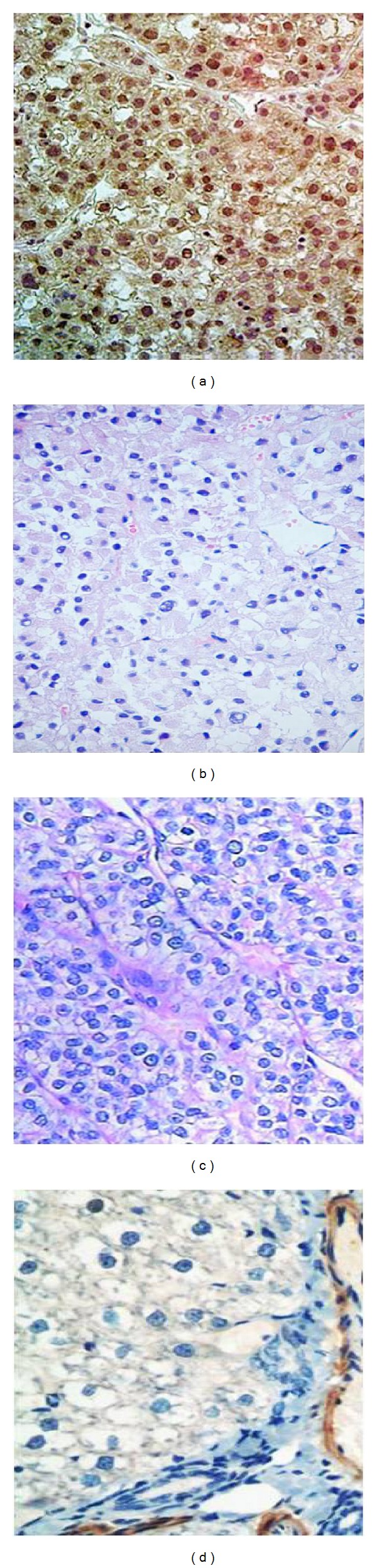
Representative images showing the presence of SALL4 protein in liver cancer and noncancerous tissue sections. (a) The positive expression of SALL4 in hepatocellular carcinoma tissues (the scoring is 2+); (b) the negative expression of SALL4 in liver tissues with chronic hepatitis B virus; (c) the negative expression of SALL4 in liver samples with liver cirrhosis; and (d) the negative expression of SALL4 in liver samples of health controls. (400x magnification).

For the expression of SALL4 in HCC tissues, strong nuclear staining in more than 1% of the tumor area was regarded as positive for SALL4. All tissue sections were analyzed and scored independently by three experienced pathologists. The expression of SALL4 was semiquantitatively analyzed by an immunohistochemical score combined with the percentage of hepatic cells showing specific immunoreactivity. The scoring system was as follows: the percentage of positively staining cells was graded as 0 (no staining), 1+: >0 and ≤25% of cells positive, 2+: >25 and ≤50% of cells positive, 3+: >50 and ≤90% of cells positive, 4+: >90% of cells positive. These results were reported as positive (score = 1, 2, 3, 4) or negative (score = 0) (Figure 1).

### 2.3. Enzyme-Linked Immunosorbent Assay for SALL4 Serum Levels Analysis

All 201 sera samples used in the study were collected into anticoagulant containing tubes at the time of diagnosis, centrifuged, cell-free supernatants separated, and stored at −80°C until testing. Detection of SALL4 serum levels was performed by two researchers independently. Commercial ELISA kits (Huamei Biotechnology Co., Ltd., Wuhan, China) were available for serum SALL4 serum detection in the study. All steps were done according to the manufacturer's instruction. Briefly, standard and experimental samples (pretreated with sample diluents) were added to an individual well of 96 wells microplates, coated with anti-human SALL4 monoclonal antibodies for 2 hours at 37°C. Then the liquid of each well was removed and further incubated with biotin-antibody for 1 hour at 37°C. After washing, the wells were incubated with HRP-avidin for 1 hour at 37°C. In the final step, the color reaction was developed using the TMB substrate. After blocking the reaction with stop solution, the OD values for all wells were read at a wavelength of 450 nm on a microplate reader, and then the OD value and concentration of SALL4 antigen (U/mL) of each well were calculated using standards curves. When the concentration of SALL4 was less than 37.5 pg/mL (the lowest limit of the standard curve), the value was set as equal to zero. Each sample was tested in duplicate.

### 2.4. Statistical Analysis

The statistical analyses were done with SPSS software (version 16.0). Categorical variables were expressed as percentages. For immunohistochemistry, the Fisher exact test and the Student's* t*-tests were used, to evaluate the significance between two samples involved in the analysis, as indicated. To test the difference between two independent groups, we used also Mann-Whitney *U* test. Probability value less than 0.05 was considered statistically significant.

## 3. Results

### 3.1. Expression Analysis of SALL4 in HCC Tissues by Immunohistochemistry

For 38 HCC tissues, 18/38 (47.4%) showed staining of SALL4 and the scores were 1+ (9/38, 23.7%), 2+ (5/38, 13.2%), 3+ (4/38, 10.5%), respectively. The association between SALL4 expression and the clinicopathologic characteristics is showed in Table 2. We found that the expression of SALL4 has no relation with any of the clinicopathologic characteristics assessed (age, sex, histologic grade, tumor locality, vascular invasion, tumor size, nodal metastasis, TNM stage, and so on). The expression of SALL4 was not visualized in nonneoplastic liver samples.

### 3.2. The Serum SALL4 Levels in HCC and Three Controls

**Figure 2:**
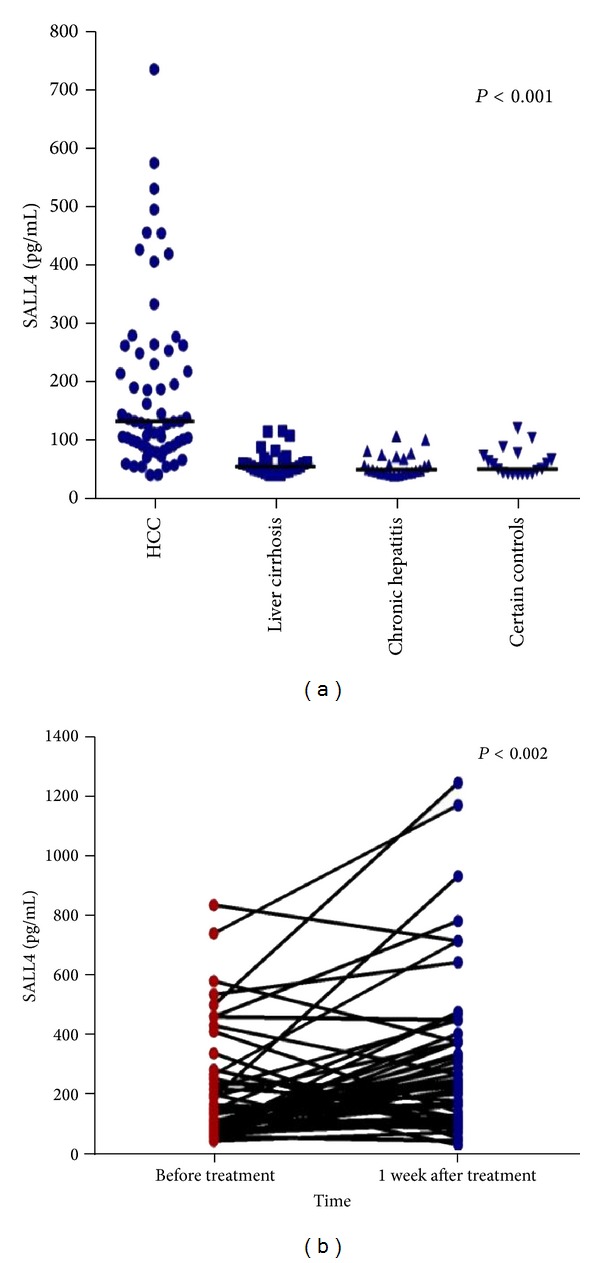
(a) The serum SALL4 levels in HCC and three controls. The concentration of serum SALL4 in HCC was much higher than that in controls (*P* < 0.001). The three control groups had no significant differences. (b) The comparison of serum SALL4 levels (*n* = 52) before and after treatment. The level of serum SALL4 after treatment was higher than before treatment (*P* < 0.002).

We further detected the serological levels of SALL4 from 127 patients with HCC, 27 patients with chronic hepatitis, 24 patients with liver cirrhosis, and 23 normal controls. The SALL4 positive sera from HCC patients were 64/127 (50.4%), with significantly higher values as compared with all three controls. No significant differences were detected between the three control groups (as Table 3 and Figure 2). In addition, the Spearman correlation analysis proved that the serological presence of SALL4 had no relationship with serum AFP (*r* = 0.005, *P* = 0.639).

### 3.3. Analysis of the SALL4 Positive Serum from HCC Patients before and after Treatment

Next, we had followed up these 64 HCC patients with SALL4 positive serum for two years to determine the relationship with tumor recurrence and overall survival. From these 64 HCC patients, 12 HCC patients did not receive any treatment. The rest of 52 HCC patients received surgery (31/52) or TACE (21/52) treatment afterwards. Then we analyzed the SALL4 serum levels of these HCC patients before treatment and after treatment 1 week later. The statistics analysis showed that the concentration of SALL4 after treatment within one week reached a peak (median 229.46 pg/mL, IQR 117.17–371.83) (as shown in Figure 2(b)), which had a significant difference for SALL4 serum levels before treatment (median 134.43 pg/mL, IQR 90.66–257.91, *P* < 0.002). Furthermore, 40 serum samples from these 52 HCC patients were collected after 7 days and 30 days after treatment. We then discovered that the median concentration of serum SALL4 of these patients reached the maximum level after one week after treatment and dropped quickly after one month (median 80.12 pg/mL, IQR 48.07–136.48, *P* < 0.0001) (as shown in Table 4).

### 3.4. The Relationship between Serum SALL4 Expression and Tumor Recurrence and Prognosis of Patients in HCC

As above described, these 64 HCC patients with SALL4 positive serum were followed up for two years. During the follow-up these 64 HCC patients, 8 of total 64 HCC patients were out of touch. From remaining 56 HCC patients, 28/56 (50%) showed tumor recurrence. According to the median level of SALL4 serum in HCC, we divided 56 patients into two groups. The high-SALL4 group (≥30.43 ng/mL, *n* = 29) had serum SALL4 concentration that was equal to or exceeded the median level of SALL4 values, whereas low-SALL4 group (<130.43 ng/mL, *n* = 27) had SALL4 serum concentration that was lower than the median level of SALL4 values. We then discovered that the high-SALL4 patients group had a recurrence of 19/29 (65.5%) while the low-SALL4 group had only 9/27 (33.3%) and the difference was significant (*P* < 0.016) (as shown in Table 5).

**Figure 3:**
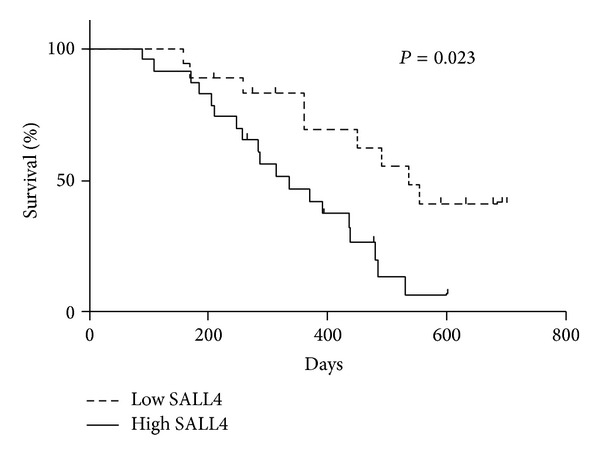
Kaplan-Meier curves showed a lower rate of overall survival in HCC patients with high expression of serum SALL4 than with low expression of serum SALL4.

Furthermore, we carried out overall survival analysis (Kaplan-Meier analysis) for these 56 HCC patients. The analysis showed HCC patients with high SALL4 serum levels had worse prognosis than patients with low serum SALL4 expression (*P* = 0.013) (as shown in Figure 3), which means HCC patients with low serum concentration of SALL4 protein had a better prognosis. The median survival of patients with SALL4 low serum levels was of 536 days, whereas the median survival of patients with high SALL4 serum levels was only 375 days (*P* = 0.023).

## 4. Discussion 

Hepatocellular carcinoma (HCC) is one of the most common malignant cancers in the world and is often associated with poor prognosis [15]. Tumor recurrence and metastasis are major obstacles to the long-term survival of HCC patients. Early intervention with treatment is very important to obtain survival benefit. The better understanding of biology molecular events in the HCC recurrence and metastasis is critically needed. Here, we report that high SALL4 serum levels are a potential novel biological biomarker correlating with tumor recurrence and poor survival of the HCC patients.

SALL4, a transcription factor that plays an essential role in the embryonic development and self-renewal of embryonic stem (ES) cells, was detected and proved to have a very high expression in leukemia and myelodysplastic syndrome [16–19]. Some investigators had confirmed that SALL4 is also overexpressed in cells from breast cancer, Yolk Sac tumor, non-small cell lung carcinomas (NSCLC), and testicular germ tumors [20–23]. Some researchers have verified that SALL4, a protein expressed in fetal, but not adult, liver cells, was reexpressed in liver tissues of a subgroup of adult hepatocellular carcinomas, which is often associated with bad prognosis [14]. Yong et al. and Lee et al. proposed that SALL4 was related with hepatocellular carcinoma with poor prognosis and, furthermore, could be used as a potential target protein for therapeutic purposes [15, 24].

It has been shown that SALL4 has functional role(s) in metastasis and drug resistance in aggressive endometrial cancer [25]. As a consequence of its functional roles in cancer cell and absence in adult normal tissue, SALL4 is both a marker for an aggressive subtype of hepatocellular carcinoma (HCC) and a potential target to prevent the high-risk occurrence of endometrial cancer [14]. In the study, we studied the expression of SALL4 in HCC liver tissues sections and, then, correlate the relationship between clinical characteristics and SALL4 tissue expression. Our studies showed that SALL4 liver tissue expression was not associated with any of the clinical characteristics assessed (such as histologic grade, tumor locality, and vascular invasion/metastasis). Furthermore, our results are in disagreement with previous report. Another study proposed that SALL4 tissue expression was associated with older age, male sex, intestinal-type histology, and synchronous hepatic metastasis in gastric carcinoma [26]. The data from another study also emphasized SALL4 expression was completely negative in hepatocellular carcinoma [26]. Meanwhile, there is a study reporting that the expression of SALL4 does not rule out secondary somatic malignancies such as HCCs, which is almost comparable to our study [27]. Collectively, these data indicated that SALL4 expression in tumor tissues has high degree of tumor selectivity and complexity.

Usually these studies about the role of SALL4 were focused on tumor cell lines and tumor tissues investigating carcinogenesis and treatment [28, 29]. As we know, cell culture studies do not directly address the role of the tumor microenvironment and that cellular responses (and the genes that control these responses) may differ from cell line to cell line. Although the role of SALL4 was evaluated in HCC cell lines and it has been considered as a valuable biomarker and also as potential therapeutic target, the reports relating the SALL4 serological levels in HCC patients are scarce, especially in association with overall survival of HCC patients. In the study, our results suggested that SALL4 serum concentrations in HCC patients were significantly higher, as compared with the three control groups (*P* < 0.001). The SALL4 serum levels reached a maximum concentration after one week after treatment and dropped dramatically after one month. In order to further evaluate the role of SALL4 serum levels in the prognosis of HCC patients, we interrelated the relationship between SALL4 serum concentrations, tumor recurrence, and metastasis, as well as overall survival rate of HCC patients. To perform these analyses, we divided 56 HCC patients into high and low groups, according to SALL4 serum level. Interestingly, our results for the first time indicated that HCC patients with high SALL4 serum levels had poor prognosis, proved by both tumor recurrence and poor overall survival rate. In fact, it is still unclear how SALL4 levels are regulated and which target genes are directly activated by SALL4 transcription factor in normal hepatic cells. Thus, the role of SALL4 in cancer is still controversial, while its function in HCC has not yet been determined [30].

In the study, we examined the expression of SALL4 in HCC tissues and serum and its clinical prognostic significance. 50.4% HCC patients were detected to have higher serum SALL4 level than control groups and the concentration of serum SALL4 reached top after treatment within one week and dropped quickly one month later. Taken together, these HCC patients with higher serum SALL4 expression had poor prognosis both in tumor recurrence and overall survival. To our knowledge, the study for the first time related the SALL4 serological levels in HCC patients especially in association with overall survival of HCC patients. Although the patient population in the study is limited, our findings are clinically significant, opening a novel avenue for further investigation of molecular events governing the pathogenesis of HCC and its metastatic modality. Further studies are required to understand the relationship between high SALL4 serum levels and their direct malignancy involvement in hepatic cells.

## 5. Conclusion

High serum levels of SALL4 protein could be used as a novel biomarker predicting tumor recurrence and poor survival rate of patients afflicted with HCC.

## Figures and Tables

**Table 1:** Clinicopathologic characteristics of HCC patients.

Age (y)	
Mean ± SD	52.65 ± 12.47
Sex %	
Male	86.8 (33/38)
Female	13.2 (5/38)
Tumor focality %	
Solitary	89.5 (34/38)
Multiple	10.5 (4/38)
Tumor size (cm)	
Mean ± SD	6.18 ± 2.89
Histologic grade %	
Well differentiated	7.9 (3/38)
Moderately differentiated	71.1 (27/38)
Poor differentiated	21 (8/38)
Nodal metastasis	
Present	15.8 (6/38)
Absent	84.2 (32/38)
Vascular invasion	
Present	31.6 (12/38)
Absent	68.4 (26/38)
Clinical T stage %	
T_1_	21.1 (8/38)
T_2_	21.1 (8/38)
T_3_	39.5 (15/38)
T_4_	18.3 (7/38)

**Table 2:** Association between SALL4 expression and clinicopathologic characteristics in HCC.

Variables	SALL4 expression		*P*
	Positive	Negative	
	(score = 1–4)	(score = 0)	
	*n* = 18 (%)	*n* = 20 (%)	
Age (y)	50.83 ± 10.33	54.31 ± 14.19	0.392
Sex			
Male	17 (94)	18 (90)	1.000
Female	1 (6)	2 (10)	
Tumor size (cm)	5.44 ± 2.71	6.67 ± 3.01	0.135
Histologic grade			
Well differentiated	3 (17)	0 (0)	0.101
Moderately differentiated	13 (72)	14 (70)	
Poor differentiated	2 (11)	6 (30)	
Tumor focality			
Solitary	15 (83)	19 (95)	0.328
Multiple	3 (17)	1 (5)	
Nodal metastasis			
Present	2 (11)	4 (20)	0.663
Absent	16 (89)	16 (80)	
Vascular invasion			
Present	4 (22)	8 (40)	0.307
Absent	14 (78)	12 (60)	
T-grade			
T_1_-T_2_	11 (61)	6 (30)	0.101
T_3_-T_4_	7 (39)	14 (70)	
Stage			
I-II	11 (61)	14 (70)	0.734
III-IV	7 (39)	6 (30)	

**Table 3:** The serum SALL4 levels in HCC and controls (pg/mL).

Variables	Median	IQR	*P*
HCC	130.43	89.40–258.47	<0.001
Chronic hepatitis	53.19	44.96–67.12	
Liver cirrhosis	48.20	42.77–69.22	
Certain controls	48.58	40.99–71.29	

HCC: hepatocellular carcinoma.

**Table 4:** The serum levels of SALL4 of 40 HCCs before and after treatment (pg/mL).

Variables	Median	IQR	*P*
Before treatment	152.54	103.12–277.26	<0.0001
1 week after treatment	220.42	118.64–371.06	
1 month after treatment	80.12	48.07–131.72	

**Table 5:** The relationship between SALL4 expression and tumor recurrence.

Tumor recurrence	Number of each group		*P*
	Low SALL4 group	High SALL4 group	
	(<130.43 pg/mL)	(≥130.43 pg/mL)	
Absent	18	10	0.016
Present	9	19	
